# Diagnostic accuracy of early urinary index changes in differentiating transient from persistent acute kidney injury in critically ill patients: multicenter cohort study

**DOI:** 10.1186/cc12582

**Published:** 2013-03-26

**Authors:** Bertrand Pons, Alexandre Lautrette, Johanna Oziel, Jean Dellamonica, Régine Vermesch, Eric Ezingeard, Christophe Mariat, Gilles Bernardin, Fabrice Zeni, Yves Cohen, Bernard Tardy, Bertrand Souweine, François Vincent, Michael Darmon

**Affiliations:** 1 Medical ICU, Saint-Etienne University Hospital, Avenue Albert Raymond, Saint-Priest en Jarez 42270, France; 2 Jacques Lisfranc Medical School, Saint-Etienne University, 15 Rue Ambroise Paré, Saint-Etienne 42000, France; 3 Medical ICU, Gabriel Montpied University Hospital, 58 Rue Montalembert, 63003 Clermont Ferrand cedex 1, France; 4 Clermont-Ferrand Medical School, Auvergne University, 28 place Henri-Dunant BP 38, 63001 Clermont-Ferrand cedex 1, France; 5 Medical-Surgical Intensive Care Unit, Avicenne University Hospital, APHP, 125 rue de Stalingrad, Bobigny 93009, France; 6 Medical Intensive Care Unit, Archet University Hospital, 151 Rte Saint Antoine Ginestiere, Nice 06202, France; 7 Nephrology, Dialysis and Renal Transplantation, Saint-Etienne University Hospital, Avenue Albert Raymond, Saint-Priest en Jarez 42270, France; 8 Nice University, UFR de Médecine, 28 Avenue de Valombrose, 06107 NICE cedex 2, France; 9 Bobigny Medical School, Paris-13 University, 74 rue Marcel Cachin, 93017 Bobigny cedex, France; 10 Thrombosis Research Group, EA 3065, Saint-Etienne University Hospital, Avenue Albert Raymond, Saint-Priest en Jarez 42270, France; 11 Department of Emergency Medicine, Saint-Etienne University Hospital, Avenue Albert Raymond, Saint-Priest en Jarez 42270, France

## Abstract

**Introduction:**

Urinary indices have limited effectiveness in separating transient acute kidney injury (AKI) from persistent AKI in ICU patients. Their time-course may vary with the mechanism of AKI. The primary objective of this study was to evaluate the diagnostic value of changes over time of the usual urinary indices in separating transient AKI from persistent AKI.

**Methods:**

An observational prospective multicenter study was performed in six ICUs involving 244 consecutive patients, including 97 without AKI, 54 with transient AKI, and 93 with persistent AKI. Urinary sodium, urea and creatinine were measured at ICU admission (H0) and on 6-hour urine samples during the first 24 ICU hours (H6, H12, H18, and H24). Transient AKI was defined as AKI with a cause for renal hypoperfusion and reversal within 3 days.

**Results:**

Significant increases from H0 to H24 were noted in fractional excretion of urea (median, 31% (22 to 41%) and 39% (29 to 48%) at H24, *P *< 0.0001), urinary urea/plasma urea ratio (15 (7 to 28) and 20 (9 to 40), *P *< 0.0001), and urinary creatinine/plasma creatinine ratio (50 (24 to 101) and 57 (29 to 104), *P *= 0.01). Fractional excretion of sodium did not change significantly during the first 24 hours in the ICU (*P *= 0.13). Neither urinary index values at ICU admission nor changes in urinary indices between H0 and H24 performed sufficiently well to recommend their use in clinical setting (area under the receiver-operating characteristic curve ≤0.65).

**Conclusion:**

Although urinary indices at H24 performed slightly better than those at H0 in differentiating transient AKI from persistent AKI, they remain insufficiently reliable to be clinically relevant.

## Introduction

Acute kidney injury (AKI) affects approximately 5 to 30% of critically ill patients and remains associated with high mortality rates [1-3]. AKI not due to urinary tract obstruction is usually described as either transient or persistent. Transient AKI is due to low renal perfusion and is promptly reversible after normalization of the hemodynamic parameters, whereas persistent AKI is believed to be due to renal tubular damage or dysfunction [4-6]. Distinguishing transient AKI from persistent AKI may help to optimize treatment decisions in patients with AKI. Urinary indices such as the fractional excretion of sodium (FeNa) and the fractional excretion of urea (FeUrea) are believed to be reliable for separating transient AKI from persistent AKI [4,5,7-9]. However, these indices may be affected by diuretic agents or sepsis [10]. In addition, little information is available on their performance for separating transient AKI from persistent AKI in critically ill patients [11]. Urine or plasma biomarkers such as neutrophil gelatinase-associated lipocalin and IL-18 have been evaluated as tools for distinguishing between transient AKI and persistent AKI [12-15] but produced conflicting results and therefore cannot be recommended for widespread use at present [13,16-18].

Hemodynamic and renal perfusion changes have been shown to affect sodium excretion during the first 24 hours of sepsis in a sheep model [19]. Sepsis-associated changes in urinary indices promptly returned to normal after renal function recovery in these animals [19]. The time-course of the urinary indices may thus differ between patients with transient AKI and persistent AKI, and evaluating changes over time may therefore improve the urinary index performance in separating these two mechanisms of AKI.

The primary objective of this study was to evaluate the diagnostic value of changes over time of the usual urinary indices in separating transient AKI from persistent AKI. The secondary objectives were to evaluate the diagnostic value of the usual urinary indices at ICU admission and at 24 hours (H24) in separating transient AKI from persistent AKI.

## Materials and methods

### Patients

The study was approved by the institutional review board of the French Society for Intensive Care Medicine (SRLF-CE-11-326), which waived the need for signed informed consent, given the observational study design. Patients and their next of kin were informed, and none refused to participate in the study.

Six university-hospital ICUs in France participated in the study between June 2011 and April 2012. Consecutive adults admitted to these ICUs were included in the absence of obstructive renal disease, renal replacement therapy (RRT) for chronic renal dysfunction, and pregnancy. Patients for whom urine could not be collected according to the study protocol were excluded secondarily; as were patients with hospital stays shorter than 72 hours, since they could not be classified as having transient AKI or persistent AKI according to our definitions.

### Definitions

AKI was defined according to the Acute Kidney Injury Network classification scheme as a plasma creatinine level increase ≥26.4 μmol/l, a plasma creatinine increase ≥150% from baseline, or urine output < 0.5 ml/kg/hour for ≥6 hours [20]. For patients whose baseline plasma creatinine level was unknown, this variable was estimated using either the plasma creatinine nadir during the ICU stay or, in patients who died before AKI resolution, the Modification of Diet in Renal Disease formula [6,21,22].

Transient AKI was defined as AKI with a cause of renal hypoperfusion according to the attending physician and recovery within 3 days [6,11]. Recovery of AKI was defined as reversal of the oliguria (in the absence of diuretic treatment) and/or ≥50% decrease in plasma creatinine and/or a return of plasma creatinine to the baseline value (whether measured or estimated using the Modification of Diet in Renal Disease formula) [11,23]. For patients having both oliguria and plasma creatinine changes defining AKI, both correction of plasma creatinine and oliguria were required to define recovery. Persistent AKI was defined as renal dysfunction without recovery within 3 days. Oliguria was defined as urine output < 0.5 ml/kg/hour for ≥6 hours [11,23].

Diuretic use at every time of this study was defined as use of diuretics in the 6 hours preceding urinary indices measurement.

Changes in urinary indices between ICU admission (H0) and H24 are reported as the ratio between urinary indices at H0 by urinary indices at H24

### Data collection

Each patient was assessed during the first 6 hours following ICU admission. Plasma sodium, urea, and creatinine levels were collected at ICU admission and then on days 1, 2, and 3. Urinary sodium, potassium, urea, and creatinine were measured at H0 and then on four consecutive 6-hour urine samples (H6, H12, H18, and H24). Urinary/plasma (U/P) ratios were used to calculate FeNa and FeUrea as follow:

$$\begin{array}{c} \text{FeNa}\;\left(\%\right)=\left(\left[\text{U}/\text{P}\;\text{sodium}\right]\;/\;\left[\text{U}/\text{P}\;\text{creatinine}\right]\right)\times 100\\ \text{FeUrea}\;\left(\%\right)=\left(\left[\text{U}/\text{P}\;\text{urea}\right]\;/\;\left[\text{U}/\text{P}\;\text{creatinine}\right]\right)\times 100 \end{array}$$

Plasma sodium, creatinine, and urea concentrations used for the calculations were those obtained closest in time to the urine sample.

The Logistic Organ Dysfunction score, the Simplified Acute Physiology Score version II and the Knaus scale score were calculated at ICU admission [24-26]. Sepsis was diagnosed using the criteria developed at the American College of Chest Physicians/Society of Critical Care Medicine consensus conference [27]. Individual organ failure was defined as a Logistic Organ Dysfunction score > 1 point for the relevant system, except the kidney [24]. Diuretic use was defined as the use of diuretics at any time during the first 24 hours in the ICU.

### Statistical analysis

Changes, as percentages, are described as the mean ± standard deviation and other data as the median (interquartile range) or number (percentage). Categorical variables were compared using Fisher's exact test and continuous variables using the nonparametric Wilcoxon test, Mann-Whitney test, or Kruskal-Wallis test. The Friedman test was used to compare continuous variables across the three patient groups (no AKI, transient AKI, and persistent AKI) and to evaluate changes in urinary index values over time.

To assess the performance of urinary indices or their changes over the first 24 hours in distinguishing transient AKI from persistent AKI, we plotted the receiver-operating characteristic curves for the proportion of true positives against the proportion of false positives, depending on the prediction rule used to classify patients as having persistent AKI. The same strategy was used to assess the performance of indices and their changes over time in two predefined patient subgroups; namely, patients who did not receive diuretic therapy and patients without sepsis.

All tests were two-sided, and *P *< 0.05 was considered statistically significant. Statistical tests were carried out using the SPSS 13 software package (IBM, Armonk, NY, USA).

## Results

### Study population

During the study period, 244 patients with a median age of 63 years (52 to 73 years) were included. Their main characteristics are reported in Table 1. According to our definitions, 97 patients had no AKI (39.8%), 54 patients (22.1%) had transient AKI, and 93 patients (38.1%) had persistent AKI.

**Table 1:** Characteristics of patients without acute kidney injury (AKI), with transient AKI, and with persistent AKI

	No AKI (*n *= 97)	Transient AKI (*n *= 54)	Persistent AKI (*n *= 93)	*P *value^a^
Patient characteristics				
Male gender	56 (57.7)	34 (62.9)	66 (71.0)	0.16
Age (years)	60 (49 to 70)	65 (52 to 74)	67 (56 to 76)	**0.02**
Knaus C or D	30 (30.9)	18 (35.3)	31 (36.0)	0.73
LOD score at admission	4 (2 to 7)	5 (3 to 8)	6 (4 to 9)	**0.006**
SAPS II score at admission	41 (31 to 54)	43 (34 to 52)	46 (36 to 59)	0.06
Baseline plasma creatinine (μmol/l)	55 (45 to 65)	56 (44 to 69)	65 (53 to 89)	**0.0006**
Risk factors for AKI				
Chronic heart failure	11 (11.3)	11 (20.3)	18 (19.4)	0.22
Chronic kidney disease^b^	2 (2.1)	1 (1.9)	12 (12.9)	**0.02**
Sepsis	36 (38.7)	32 (60.4)	48 (55.2)	**0.02**
Aminoglycosides	6 (6.7)	7 (14.6)	12 (14.0)	0.22
Ionidated contrast agents	37 (41.1)	11 (22.9)	22 (25.3)	**0.03**
Diabetes mellitus	8 (8.3)	15 (27.8)	24 (25.8)	**0.002**
Reason for ICU admission				
Acute respiratory failure	24 (24.7)	17 (31.5)	19 (20.5)	0.32
Coma	36 (37.1)	8 (14.8)	15 (16.1)	**0.0006**
Sepsis/shock	13 (13.4)	19 (35.2)	43 (46.3)	**< 0.0001**
Treatments				
Need for vasoactive drugs	80 (51.6)	25 (46.3)	55 (59.1)	0.29
Mechanical ventilation	79 (81.4)	30 (55.6)	55 (59.4)	**0.0006**
Renal replacement therapy	1 (1.0)	0	13 (14.0)	**< 0.0001**
Diuretics	30 (30.9)	17 (31.5)	33 (35.5)	0.78
Renal function at admission				
Diuresis (ml/kg/hour)	1.29 (0.69 to 1.99)	1.14 (0.73 to 2.00)	0.79 (0.48 to 1.22)	**0.0005**
Plasma urea (mmol/l)	5.9 (4.4 to 8.1)	10.8 (7.3 to 14.9)	14.1 (7.6 to 25.2)	**< 0.0001**
Plasma creatinine (μmol/l)	64 (53 to 80)	111 (76 to 170)	139 (95 to 253)	**< 0.0001**
Urinary indices				
FeNa (%)	0.7 (0.2 to 1.7)	0.8 (0.3 to 2.7)	1.1 (0.4 to 2.8)	**0.03**
FeUrea (%)	35 (24 to 45)	29 (21 to 39)	29 (21 to 36)	**0.03**
U/P urea	23 (14 to 34)	11 (7 to 22)	10 (4 to 19)	**< 0.0001**
U/P creatinine	70 (39 to 129)	37 (17 to 98)	38 (17 to 74)	**< 0.0001**
Outcome				
ICU mortality	22 (22.7)	12 (22.2)	21 (22.5)	0.99
Hospital mortality	25 (25.8)	17 (31.5)	26 (28.0)	0.56

Results reported as median (interquartile range) or *n *(%). FeNa, fractional excretion of sodium ([U/P sodium]/[U/P creatinine])×100; FeUrea, fractional excretion of urea ([U/P urea]/[U/P creatinine])×100; LOD, Logistic Organ Dysfunction score (can range from 0 to 22); SAPS, Simplified Acute Physiology Score; U/P, urinary/plasma. ^a^*P *values are for comparisons across the three patient groups. ^b^Chronic renal failure was defined as creatinine clearance before ICU admission < 60 ml/minute. Bold data represent variables that differ statistically across groups of patients.

At ICU admission, the median Simplified Acute Physiology Score version II was 44 (33 to 55) and the median LOD score was 5 (4 to 8). Most patients were admitted for medical conditions (*n *= 224, 91.9%). The main risk factors for AKI were sepsis (49.8%), exposure to contrast agents (31.1%), diabetes mellitus (19.3%), aminoglycoside therapy (10.2%), and chronic kidney disease (6.1%).

No patient was receiving RRT t the time of the study. RRT was required during the ICU stay in 21 patients (8.6%), usually during the first 24 hours in the ICU (*n *= 14/244, 5.7%).

### Urinary indices at ICU admission

**Figure 1:**
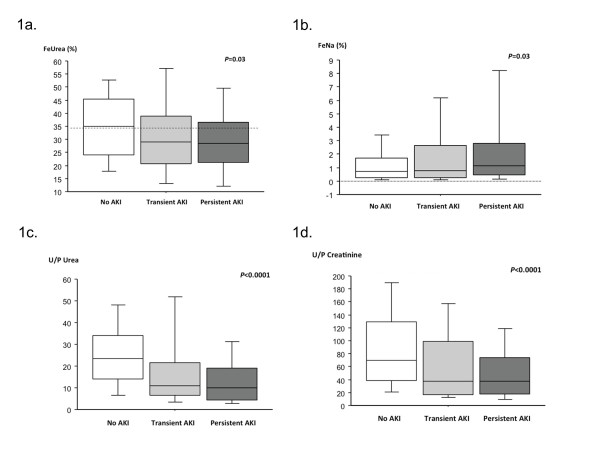
**Boxplot representation of urinary indices at baseline according to renal function. (a) **Fractional excretion of urea (FeUrea) according to renal function (*P *= 0.03). **(b) **Fractional excretion of sodium (FeNa) according to renal function (*P *= 0.03). **(c) **The urine/plasma (U/P) urea ratio according to renal function (*P *< 0.0001). **(d) **The U/P creatinine ratio according to renal function (*P *< 0.0001). AKI, acute kidney injury. Whiskers represent 5th to 95th percentiles.

Table 1 and Figure 1 report the main urinary index values at ICU admission. Median FeNa, FeUrea, and U/P ratios of urea and creatinine differed significantly across the groups with no AKI, transient AKI, and persistent AKI.

### Changes in urinary indices during the first 24 hours after ICU admission in the overall population

**Figure 2:**
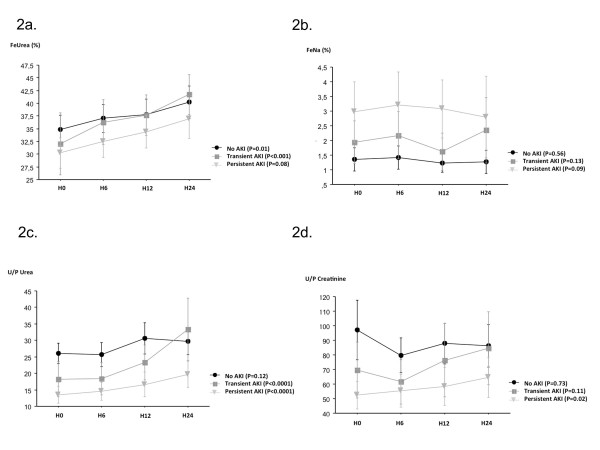
**Changes in urinary indices during the first 24 hours after ICU admission according to renal function. (a) **Fractional excretion of urea (FeUrea) according to renal function. **(b) **Fractional excretion of sodium (FeNa) according to renal function. **(c) **The urine/plasma (U/P) urea ratio according to renal function. **(d) **The U/P creatinine ratio according to renal function. AKI, acute kidney injury; H0, ICU admission; H6, H12, H18, and H24, 6-hour urine samples during the first 24 ICU hours. Data presented as mean ± 95% confidence interval.

Significant increases were noted between H0 and H24 in FeUrea (from 31% (22 to 41%) to 39% (29 to 48%), *P *< 0.0001), in U/P urea ratio (from 15 (7 to 28) to 20 (9 to 40), *P *< 0.0001), and in U/P creatinine ratio (from 50 (24 to 101) to 57 (29 to 104), *P *= 0.01) (Figure 2; Table S1 in Additional file 1). FeNa did not change significantly (*P *= 0.13) during the first 24 hours in the ICU.

### Changes in urinary indices during the first 24 hours after ICU admission in the groups without AKI, with transient AKI, and with persistent AKI

In the group with transient AKI, FeUrea increased significantly from H0 to H24 (from 29% (21 to 39%) to 42% (30 to 51%), *P *< 0.001) (Figure 2; Table S1 in Additional file 1). No significant changes in FeUrea occurred in the group with persistent AKI (*P *= 0.08). In none of the three groups did FeNa or the U/P creatinine ratio change significantly. The U/P urea ratio increased significantly in both AKI groups (transient AKI, from 11 (7 to 22) to 29 (10 to 42), *P *< 0.0001; and persistent AKI, from 10 (4 to 19) to 13 (6 to 27), *P *< 0.0001).

Changes in urinary indices in patients with and without diuretic therapy are reported in Table S2 and Figures S1 and S2 in Additional file 1.

### Performance of urinary indices and their changes in differentiating transient from persistent acute kidney injury

Table 2 reports the performance data for the urinary indices. None of the four urinary indices at ICU admission differentiated transient AKI from persistent AKI in the overall population (area under the receiver-operating characteristic curve 0.50 to 0.59) or in the subgroups without diuretic therapy or without sepsis. Compared with values at ICU admission, values at H24 and changes from H0 to H24 performed slightly better; however, their performance remained too poor to be clinically relevant (area under the receiver-operating characteristic curve ≤0.65 for all values; Table 2). The main factors associated with persistent AKI after adjustment for confounders are reported in Table S3 in Additional file 1.

**Table 2:** Diagnostic performance of and changes in urinary indices at ICU admission and after 24 hours

Patients with AKI	Overall population (*n *= 147)	Patients without diuretics (*n *= 97)	Patients without sepsis(*n *= 60)
Urinary indices at ICU admission			
FeNa	0.59 (0.49 to 0.69)	0.54 (0.41 to 0.66)	0.59 (0.42 to 0.75)
FeUrea	0.50 (0.41 to 0.60)	0.53 (0.41 to 0.65)	0.64 (0.49 to 0.79)
U/P urea	0.57 (0.48 to 0.67)	0.56 (0.45 to 0.68)	0.59 (0.45 to 0.74)
U/P creatinine	0.55 (0.45 to 0.65)	0.53 (0.41 to 0.65)	0.61 (0.46 to 0.77)
Urinary indices at H24			
FeNa	0.61 (0.51 to 0.71)	0.64 (0,52 to 0,76)*	0,56 (0,40 to 0.72)
FeUrea	0.51 (0.40 to 0.62)	0.57 (0.43 to 0.70)	0.56 (0.39 to 0.72)
U/P urea	0.65 (0.55 to 0.75)*	0.65 (0.53 to 0.76)*	0.67 (0.51 to 0.83)*
U/P creatinine	0.58 (0.48 to 0.69)	0.56 (0.43 to 0.69)	0.58 (0.41 to 0.76)
Changes in urinary indices between H0 and H24			
FeNa	0.59 (0.49 to 0.70)	0.58 (0.45 to 0.71)	0,59 (0,40 to 0.78)
FeUrea	0.57 (0.47 to 0.68)*	0.58 (0.44 to 0.72)	0.61 (0.43 to 0.78)
U/P urea	0.61 (0.51 to 0.71)*	0.60 (0.47 to 0.73)	0.65 (0.48 to 0.81)
U/P creatinine	0.51 (0.49 to 0.62)	0.52 (0.40 to 0.65)	0.53 (0.33 to 0.70)

Diagnostic performance of urinary indices at ICU admission and after 24 hours, and changes in urinary indices over the first 24 hours in the ICU for separating transient from persistent acute kidney injury. Data are areas under the receiver-operating characteristic curves (± 95%). AKI, acute kidney injury; FeNa, fractional excretion of sodium ([U/P sodium]/[U/P creatinine])×100; FeUrea, fractional excretion of urea ([U/P urea]/[U/P creatinine])×100; H0, ICU admission; H24, 24 hours; U/P, urinary/plasma. *P < 0.05. Bold data represent AUC that differ statistically from 0.5.

## Discussion

To the best of our knowledge, this is the first study evaluating the time-course of standard urinary indices (FeNa, FeUrea, U/P urea, and U/P creatinine) in critically ill patients without AKI, with transient AKI, and with persistent AKI. The indices were not effective in differentiating transient AKI from persistent AKI. Most of them changed significantly over the first 24 hours in the ICU, but these changes did not accurately separate transient from persistent AKI.

Urinary index values at ICU admission performed poorly for differentiating transient and persistent AKI. Although many publications advocate the use of these indices, few of them focus on critically ill patients or patients with sepsis [4,5,7,8]. In addition, most of the available studies are single-center case series or retrospective cohort studies [28-31]. The definitions of AKI and transient AKI varied across studies, and most definitions of transient AKI relied on subjective criteria [28-31]. Finally, these studies included patients who did not have critical illnesses [9,31,32]. In our study, we used an objective definition of transient AKI. Most of the study patients had sepsis at ICU admission, a condition associated with alterations in renal handling of sodium and water despite normal renal perfusion or adequate fluid therapy [19]. This point may explain the differences between our results and those of previous studies [9,28,30-32]. Interestingly, FeNa and FeUrea were usually decreased in both transient AKI and persistent AKI [11,19], suggesting partial preservation of tubular function even in patients with persistent AKI [19,23]. In critically ill patients, therefore, urinary indices may be unable to distinguish transient AKI from persistent AKI.

The main objective of this study was to evaluate the diagnostic performance of urinary index changes over the first 24 hours in the ICU for separating transient AKI and persistent AKI. In sheep, FeNa declined within the first few hours after the induction of septic AKI and then returned to normal during renal recovery [19]. Similarly, urinary indices returned to normal within 24 hours in our patients, and the improvement was more rapid in the transient AKI group than in the persistent AKI group. As a result, H24 values performed significantly better than H0 values in differentiating transient AKI from persistent AKI. Nevertheless, performance of the H24 values remained too low to be clinically relevant.

Interestingly, diuretic therapy had no effect on urinary indices or their changes over time in our study. In particular, FeNa changes over the first 24 hours were not significantly different in the groups with and without diuretic therapy. Resistance to diuretics may explain this finding. Heart failure, renal hypoperfusion related to shock or sepsis, and hypoalbuminemia are common in ICU patients and can result in resistance to diuretics [33,34]. In patients given diuretics before ICU admission, rebound sodium retention, a post-diuretic effect, and diuretic braking also contribute to diuretic resistance [35,36]. Last, our patients received diuretics as bolus injections. Using continuous infusions [37,38] or higher dosages might have produced different results.

Our study has several limitations. First, our definition of transient AKI was based on renal function recovery. Indeed, a more selective definition of pre-renal AKI would have required the use of highly subjective criteria such as clinical history, physical examination, and physician judgment [9,10]. We elected to use a definition that relied on an objective criterion. In addition, our definition takes into account the continuum that probably exists between renal hypoperfusion and kidney damage [6]. The difference between our definition of transient AKI and that used in earlier studies may contribute to the discrepancies in the results. Moreover, our definition was highly sensitive for detecting patients with transient AKI, since none of these patients required RRT, but also lacked specificity, as less than 20% of patients with persistent AKI required RRT. This limitation must be taken into account when interpreting our results. In addition, an imbalance of the case mix across the three groups of patients (no AKI, transient AKI and persistent AKI) was observed. Patients without AKI were thus more frequently admitted for neurological dysfunction than patients with AKI usually admitted with severe sepsis or septic shock. This may explain the higher rate of mechanical ventilation in patients without AKI than patients with AKI and the absence of association between mortality and AKI in this study. However, performance of urinary indices in this study is consistent with previous studies performed in different ICUs or in populations with a different case mix [11,15]. Another limit of our study was that neither fluid balance nor fluid therapy was recorded. We may suppose that both these variables could influence the final results.

Although our study suggests that none of the usual urinary indices or their changes may reliably help in distinguishing transient AKI from persistent AKI, further studies are needed to assess influence of fluid challenge and fluid balance on urinary index course or performance. Last, we did not evaluate newly described biomarkers for diagnosing transient AKI. Future studies should compare these biomarkers with the standard urinary indices evaluated in our study.

## Conclusion

This study confirms the poor performance of standard urinary indices at ICU admission for differentiating transient AKI from persistent AKI in unselected critically ill patients. Although changes over the first 24 hours and values at H24 performed slightly better than values at admission, their performance remained too low to be clinically useful. Additional studies are needed to identify means of reliably separating transient and persistent AKI, with the goal of improving early therapeutic interventions.

## Key messages

• Standard urinary index values at ICU admission performed poorly for differentiating transient AKI and persistent AKI.

• Although changes over the first 24 hours and values at H24 performed slightly better than values at admission, their performance remained too low to be clinically useful.

• Performance of newest biomarkers in this setting remains to be evaluated.

## Abbreviations

AKI: acute kidney injury; FeNa: fractional excretion of sodium; FeUrea: fractional excretion of urea; RRT: renal replacement therapy; U/P: urinary/plasma.

## Competing interests

The authors declare that they have no competing interests.

## Authors' contributions

BP participated in the study concept and design, acquisition of data, analysis and interpretation of data, drafting of the manuscript and critical revision of the manuscript. AL participated in the study design, acquisition of data, analysis and interpretation of data, drafting of the manuscript and critical revision of the manuscript. JO, JD, RV, EE, CM, GB, FZ, YC and BT participated in acquisition of data, interpretation of data, and critical revision of the manuscript. BS and FV participated in the study design, acquisition of data, analysis and interpretation of data, drafting of the manuscript and critical revision of the manuscript. MD conceived the study, participated in study design and coordination, acquisition of data, statistical analysis and interpretation of data, drafting of the manuscript and critical revision of the manuscript. MD had full access to all of the data in the study and takes responsibility for the integrity of the data and the accuracy of the data analysis. All authors read and approved the final manuscript.

## Supplementary Material

Additional file 1 **Table S1 showing changes in urinary indices during the first 24 hours following ICU admission and Table S2 showing the influence of diuretic therapy**. Table S3 showing factors independently associated with persistent AKI in a conditional logistic regression model. Figure S1 showing changes in urinary indices in the overall population, in patients receiving diuretic therapy during the first 24 hours following ICU admission and in patients without diuretic therapy. Figure S2 showing change in urinary indices according to renal function and use of diuretics. Click here for file
