# Supplementary material for: Diagnostic accuracy of early urinary index changes in differentiating transient from persistent acute kidney injury in critically ill patients: multicenter cohort study
Source: Crit Care. 2013 Mar 26;17(2):R56. doi: 10.1186/cc12582 (PMC3733426; doi:10.1186/cc12582)
Supplement: Additional file 1 — Table S1 showing changes in urinary indices during the first 24 hours following ICU admission and Table S2 showing the influence of diuretic therapy. Table S3 showing factors independently associated with persistent AKI in a conditional logistic regression model. Figure S1 showing changes in urinary indices in the overall population, in patients receiving diuretic therapy during the first 24 hours following ICU admission and in patients without diuretic therapy. Figure S2 showing change in urinary indices according to renal function and use of diuretics. [file cc12582-S1.DOC]

**Diagnostic Accuracy of Early Urinary Index Changes in Differentiating Transient from Persistent Acute Kidney Injury in Critically Ill Patients: Multicenter Cohort Study**

**Pons B et al.**

**Additional File 1**

**Changes in urinary indices in patients receiving diuretic therapy during the first 24 hours following ICU admission**

Overall, 80 (32.7%) patients received diuretics before or within 24 hours after ICU admission. Among them, 30 had no AKI (30.9% of patients without AKI), 17 had transient AKI (31.5% of patients with transient AKI), and 33 had persistent AKI (35.5% of patients with persistent AKI) (Table 1). Changes in urinary indices in patients with and without diuretics are reported in Table S2, Figure S1, and Figure S2.

Among patients receiving diuretic therapy, those with transient AKI exhibited a significant increase in FeUrea during the first 24 hours in the ICU (from 29% [17-32] to 37% [27-49], *P*=0.01) (Table S2). No statistically significant changes were observed in patients without AKI or in patients with persistent AKI (*P*=0.33 and 0.24 respectively). No significant changes in FeNa, U/P urea, or U/P creatinine occurred during the first 24 hours following ICU admission in patients without AKI, with transient AKI, or with persistent AKI (Table S2).

**Table S1. Changes in urinary indices during the first 24 hours following ICU admission**

|  |  |  |  |  |  |  |
| --- | --- | --- | --- | --- | --- | --- |
|  |  | **H0** | **H6** | **H12** | **H24** | ***P* value** |
| All patients (n=244) | |  |  |  |  |  |
|  | FeNa (%) | 0.8 (0.3-2.1) | 0.8 (0.3-2.3) | 0.8 (0.3-1.9) | 0.8 (0.3-1.8) | 0.13 |
|  | **FeUrea (%)** | **31 (22-41)** | **35 (23-45)** | **36 (25-47)** | **39 (29-48)** | **< 0,0001** |
|  | **U/P Urea** | **15 (7-28)** | **17 (7-28)** | **17 (8-32)** | **20 (9-40)** | **< 0,0001** |
|  | **U/P Creatinine** | **50 (24-101)** | **49 (21-97)** | **50 (24-104)** | **57 (29-104)** | **0.01** |
| No AKI (n=97) | |  |  |  |  |  |
|  | FeNa (%) | 0.7 (0.2-1.7) | 0.7 (0.3-1.7) | 0.8 (0.2-1.5) | 0.8 (0.3-1.5) | 0.56 |
|  | **FeUrea (%)** | **35 (24-45)** | **37 (25-46)** | **37 (25-47)** | **40 (32-47)** | **0.01** |
|  | U/P Urea | 23 (14-34) | 21 (15-33) | 24 (15-39) | 26 (16-44) | 0.13 |
|  | U/P Creatinine | 70 (39-129) | 68 (36-106) | 70 (41-123) | 66 (37-120) | 0.73 |
| Transient AKI (n=54) | |  |  |  |  |  |
|  | FeNa (%) | 0.8 (0.3-2.7) | 0.8 (0.3-2.6) | 0.7 (0.3-1.6) | 1 (0.3-2.1) | 0.13 |
|  | **FeUrea (%)** | **29 (21-39)** | **37 (25-47)** | **37 (28-48)** | **42 (30-51)** | **< 0,001** |
|  | **U/P Urea** | **11 (7-22)** | **13 (8-21)** | **17 (10-30)** | **29 (10-42)** | **< 0,0001** |
|  | U/P Creatinine | 37 (17-98) | 42 (19-84) | 48 (25-85) | 68 (26-114) | 0.11 |
| Persistent AKI (n=93) | |  |  |  |  |  |
|  | FeNa (%) | 1.1 (0.4-2.8) | 0.9 (0.3-3.4) | 1.1 (0.4-3.5) | 0.6 (0.3-2.1) | 0.09 |
|  | FeUrea (%) | 29 (21-36) | 32 (22-39) | 32 (24-48) | 36 (25-46) | 0.08 |
|  | **U/P Urea** | **10 (4-19)** | **10 (4-22)** | **9 (4-25)** | **13 (6-27)** | **< 0,0001** |
|  | U/P Creatinine | 38 (17-74) | 33 (16-80) | 35 (16-80) | 39 (22-93) | 0.18 |
|  |  |  |  |  |  |  |

The data are medians [IQR].

**^a^***P* values are for comparisons across the three patient groups.

AKI, acute kidney injury; FeNa, fractional excretion of sodium ([urine sodium/plasma sodium] / [urine creatinine/plasma creatinine])·100; FeUrea, fractional excretion of urea ([urine urea/plasma urea] / [urine creatinine/plasma creatinine])·100; U/P Urea, urine urea/ plasma urea; U/P Creatinine, urine creatinine/plasma creatinine

**Table S2. Changes in urinary indices during the first 24 hours after ICU admission in patients with and without diuretic therapy**

|  |  |  | **H0** | **H6** | **H12** | **H24** | ***P* value** |
| --- | --- | --- | --- | --- | --- | --- | --- |
| **Patients with diuretics (n=80)** | | | |  |  |  |  |
|  | No AKI (n=30) | |  |  |  |  |  |
|  |  | FeNa (%) | 0.6 (0.1-3.2) | 0.7 (0.2-2) | 1.2 (0.4-2.1) | 1.1 (0.8-1.9) | 0.25 |
|  |  | FeUrea (%) | 26 (18-39) | 35 (21-46) | 33 (20-44) | 38 (31-46) | 0.33 |
|  |  | U/P Urea | 20 (9-30) | 17 (9-27) | 15 (8-21) | 17 (9-24) | 0.35 |
|  |  | U/P Creatinine | 73 (27-154) | 49 (29-126) | 46 (22-69) | 44 (31-63) | 0.2 |
|  | Transient AKI (n=17) | |  |  |  |  |  |
|  |  | FeNa (%) | 0.7 (0.2-3.9) | 1.9 (0.4-4.9) | 0.8 (0.3-2.4) | 1.5 (0.3-5.9) | 0.11 |
|  |  | **FeUrea (%)** | **29 (17-32)** | **36 (28-43)** | **32 (25-38)** | **37 (27-49)** | **0.01** |
|  |  | U/P Urea | 8 (6-13) | 9 (7-11) | 15 (5-18) | 18 (7-35) | 0.14 |
|  |  | U/P Creatinine | 29 (17-80) | 30 (15-34) | 43 (21-76) | 50 (15-116) | 0.24 |
|  | Persistent AKI (n=33) | |  |  |  |  |  |
|  |  | FeNa (%) | 2 (1-5.5) | 1.6 (0.5-8.1) | 2.4 (0.9-8.1) | 1.7 (0.5-4.2) | 0.39 |
|  |  | FeUrea (%) | 31 (21-40) | 32 (21-43) | 33 (20-49) | 35 (27-46) | 0.24 |
|  |  | U/P Urea | 6 (3-12) | 6 (4-16) | 6 (3-14) | 8 (5-15) | 0.18 |
|  |  | U/P Creatinine | 30 (11-66) | 24 (8-71) | 18 (9-49) | 33 (16-67) | 0.59 |
| **Patients without diuretics (n=164)** | | | |  |  |  |  |
|  | No AKI (n=67) | |  |  |  |  |  |
|  |  | FeNa (%) | 0.7 (0.3-1.6) | 0.7 (0.3-1.5) | 0.6 (0.2-1.4) | 0.6 (0.2-1.4) | 0.12 |
|  |  | FeUrea (%) | 37 (27-46) | 37 (31-47) | 40 (30-47) | 40 (32-49) | 0.06 |
|  |  | U/P Urea | 26 (17-35) | 24 (17-35) | 32 (19-48) | 34 (23-48) | < 0.001 |
|  |  | U/P Creatinine | 70 (47-121) | 68 (41-105) | 91 (48-128) | 83 (48-150) | 0.33 |
|  | Transient AKI (n=37) | |  |  |  |  |  |
|  |  | FeNa (%) | 0.8 (0.3-1.9) | 0.7 (0.3-1.5) | 0.7 (0.2-1.4) | 1 (0.3-1.6) | 0.55 |
|  |  | FeUrea (%) | 29 (21-39) | 37 (22-48) | 39 (30-50) | 44 (32-54) | < 0.01 |
|  |  | U/P Urea | 13 (7-27) | 17 (9-28) | 21 (12-32) | 34 (14-50) | < 0.0001 |
|  |  | U/P Creatinine | 47 (19-104) | 57 (25-109) | 54 (25-87) | 77 (32-114) | 0.19 |
|  | Persistent AKI (n=60) | |  |  |  |  |  |
|  |  | FeNa (%) | 0.8 (0.3-2.2) | 0.6 (0.2-2.4) | 0.7 (0.2-1.8) | 0.5 (0.2-1.3) | 0.32 |
|  |  | FeUrea (%) | 28 (21-36) | 31 (22-39) | 32 (25-43) | 36 (23-47) | 0.36 |
|  |  | U/P Urea | 11 (5-21) | 13 (5-24) | 14 (6-29) | 17 (8-39) | < 0.0001 |
|  |  | U/P Creatinine | 47 (21-75) | 44 (19-85) | 44 (18-84) | 52 (28-103) | 0.02 |

The data are medians [IQR].

**^a^***P* values are for comparisons across the three patient groups.

AKI acute kidney injury; FeNa, fractional excretion of sodium ([urine sodium/plasma sodium] / [urine creatinine/ plasma creatinine])·100; FeUrea, fractional excretion of urea ([urine urea/ plasma urea] / [urine creatinine/ plasma creatinine])·100; U/P Urea, urine urea/ plasma urea; U/P Creatinine, urine creatinine/ plasma creatinine.

**Table S3. Factors independently associated with persistent AKI (forward conditional logistic regression). When forced in the final model, none of the urinary indices were associated with persistent AKI nor changed the final model.**

|  | **OR** | **95%CI** | **P Value** |
| --- | --- | --- | --- |
| Male gender | 2.26 | (1.19-4.30) | 0.01 |
| SAPSII score per point  Plasma creatinine at admission >157µmol/L  Sepsis as reason for admission | 1.02  5.22  2.01 | (1.005-1.04)  (2.50-10.90)  (1.00-4.05) | 0.01  <0.0001  0.051 |

(Hosmer-Lemeshow goodness of fit: k²= 4.32; P= 0.83)

**Figure S1. Changes in urinary indices [FeUrea (a), FeNa (b), U/P urea ratio (c) and U/P creatinine ratio (d)] according to renal function in patients with diuretic therapy (mean±95%CI).**

**Figure S2. Changes in urinary indices [FeUrea (a), FeNa (b), U/P urea ratio (c) and U/P creatinine ratio (d)] according to renal function in patients without diuretic therapy (mean±95%CI).**

**Figure S1**


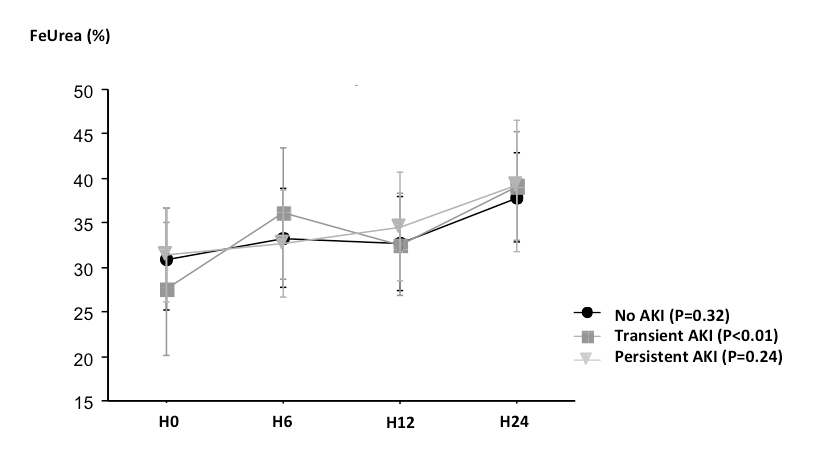

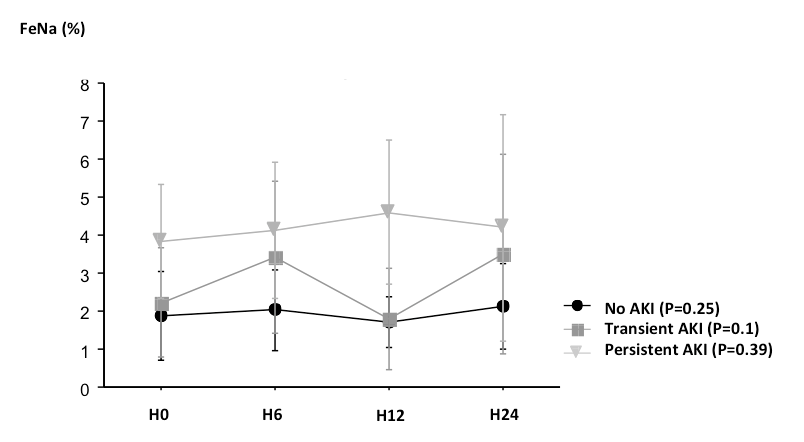
(a) (b)


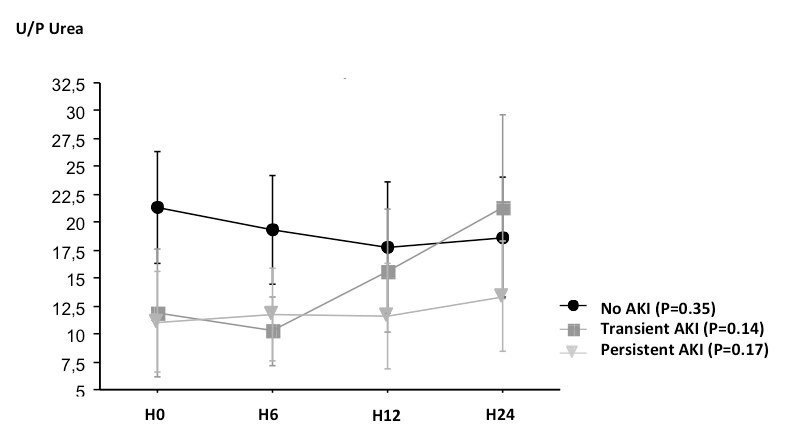


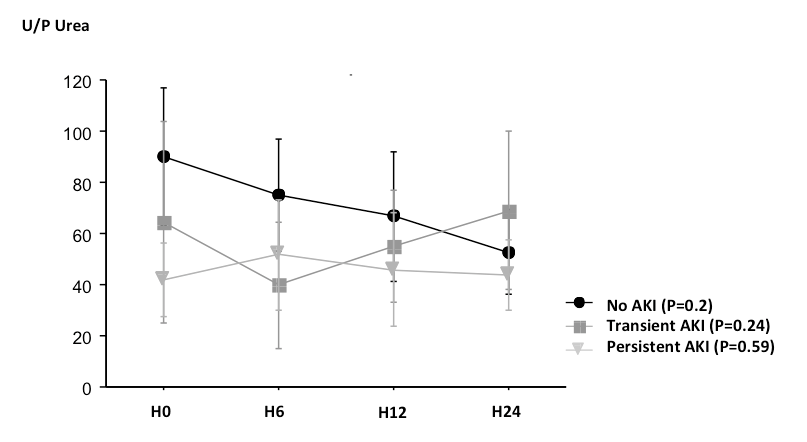
(c) (d)

**Figure S2**


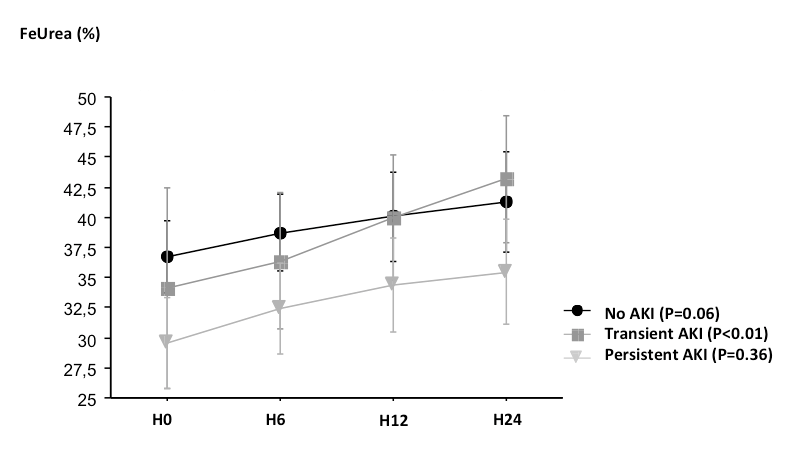

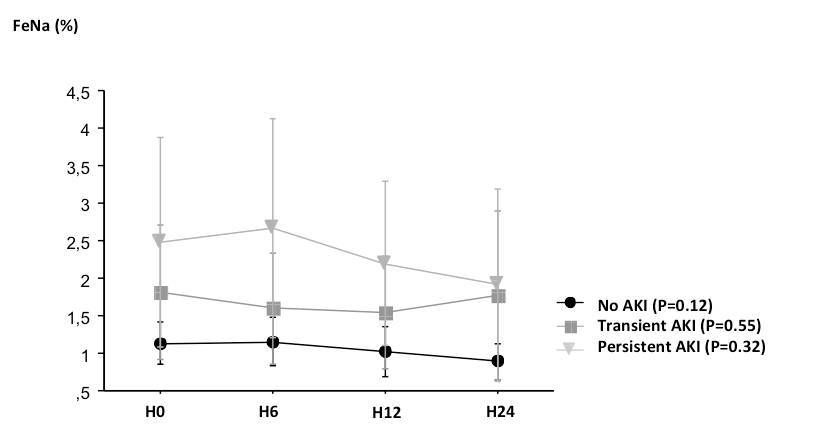
(a) (b)


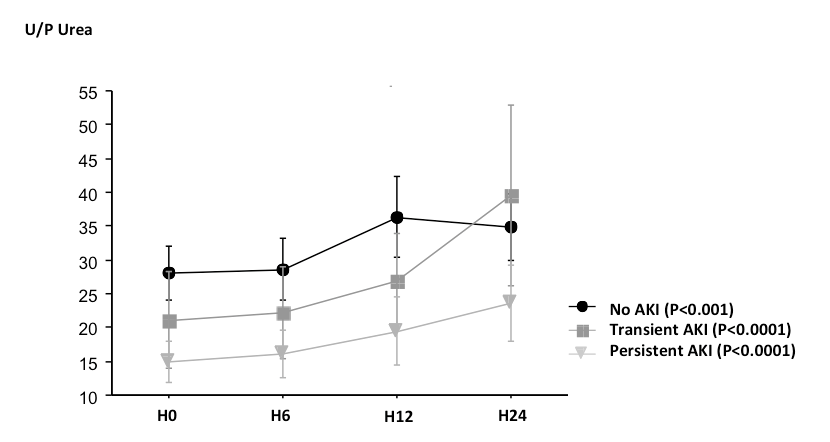

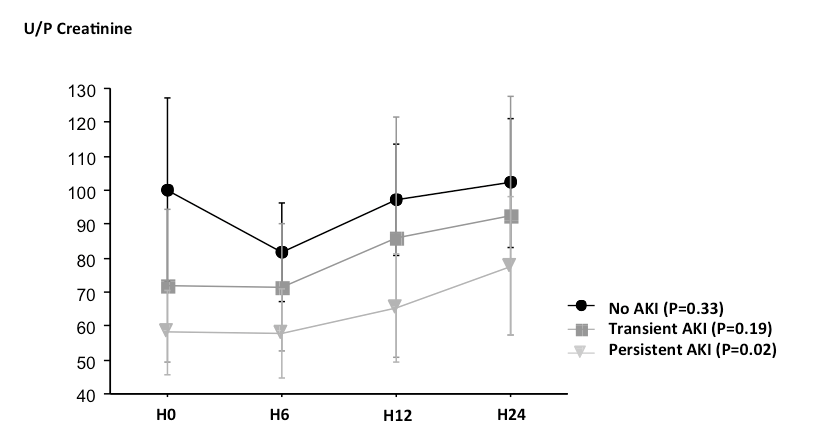


(c) (d)
